# Accelerated Development of Cervical Spine Instabilities in Rheumatoid Arthritis: A Prospective Minimum 5-Year Cohort Study

**DOI:** 10.1371/journal.pone.0088970

**Published:** 2014-02-18

**Authors:** Takashi Yurube, Masatoshi Sumi, Kotaro Nishida, Hiroshi Miyamoto, Kozo Kohyama, Tsukasa Matsubara, Yasushi Miura, Hiroaki Hirata, Daisuke Sugiyama, Minoru Doita

**Affiliations:** 1 Department of Orthopaedic Surgery, Kobe University Graduate School of Medicine, Chuo-ku, Kobe, Japan; 2 Department of Orthopaedic Surgery, Kobe Rosai Hospital, Chuo-ku, Kobe, Japan; 3 Department of Orthopaedic Surgery, Kobe Medical Center, Suma-ku, Kobe, Japan; 4 Department of Orthopaedic Surgery, Kohnan Kakogawa Hospital, Kanno-cho, Kakogawa, Japan; 5 Department of Orthopaedic Surgery, Matsubara Mayflower Hospital, Fujita, Kato, Japan; 6 Department of Evidence-Based Laboratory Medicine, Kobe University Graduate School of Medicine, Chuo-ku, Kobe, Japan; University of Texas Southwestern Medical Center, United States of America

## Abstract

**Objective:**

To clarify the incidence and predictive risk factors of cervical spine instabilities which may induce compression myelopathy in patients with rheumatoid arthritis (RA).

**Methods:**

Three types of cervical spine instability were radiographically categorized into “moderate” and “severe” based on atlantoaxial subluxation (AAS: atlantodental interval >3 mm versus ≥10 mm), vertical subluxation (VS: Ranawat value <13 mm versus ≤10 mm), and subaxial subluxation (SAS: irreducible translation ≥2 mm versus ≥4 mm or at multiple). 228 “definite” or “classical” RA patients (140 without instability and 88 with “moderate” instability) were prospectively followed for >5 years. The endpoint incidence of “severe” instabilities and predictors for “severe” instability were determined.

**Results:**

Patients with baseline “moderate” instability, including all sub-groups (AAS^+^ [VS^−^ SAS^−^], VS^+^ [SAS^−^ AAS^±^], and SAS^+^ [AAS^±^ VS^±^]), developed “severe” instabilities more frequently (33.3% with AAS^+^, 75.0% with VS^+^, and 42.9% with SAS^+^) than those initially without instability (12.9%; p<0.003, p<0.003, and p = 0.061, respectively). The incidence of cervical canal stenosis and/or basilar invagination was also higher in patients with initial instability (17.5% with AAS^+^, 37.5% with VS^+^, and 14.3% with SAS^+^) than in those without instability (7.1%; p = 0.028, p<0.003, and p = 0.427, respectively). Multivariable logistic regression analysis identified corticosteroid administration, Steinbrocker stage III or IV at baseline, mutilating changes at baseline, and the development of mutilans during the follow-up period correlated with the progression to “severe” instability (p<0.05).

**Conclusions:**

This prospective cohort study demonstrates accelerated development of cervical spine involvement in RA patients with pre-existing instability—especially VS. Advanced peripheral erosiveness and concomitant corticosteroid treatment are indicators for poor prognosis of the cervical spine in RA.

## Introduction

Rheumatoid arthritis (RA) is a chronic inflammatory disease, which affects 0.5–1% of the adult population [1]. The cervical spine is a popular focus of RA synovitis and enthesitis [2]. Rheumatoid arthritis often causes three characteristic instabilities in the cervical spine: atlantoaxial subluxation (AAS) [3]–[11], vertical subluxation (VS) of the axis [12]–[14], and subaxial subluxation (SAS) [15]. These subluxations should be noted as one of the most serious pathologies in patients with RA since they can introduce irreversible neural impairment, non-ambulation, respiratory dysfunction, or sudden death [16]. However, the prevalence of cervical spine instabilities which may lead to complications has not been fully profiled. Predictive risk factors for severe aggravation have not been comprehensively evaluated. While many studies have investigated the progression of instabilities retrospectively, limited prospective studies have been published [7], [8], [10], [17]–[22].

We previously presented a prospective multicenter cohort study for the cervical spine in established RA patients [23], [24]. The first study described the progression of instabilities, but not of lesions with impending neurological deficit [23]. The second study aimed to identify predictors for the aggravation of instabilities; however, this had limited inclusion criteria, small sample size, and did not provide conclusive evidence [24]. Thus, the hypothesis-generating data from these two studies revealed the necessity of larger-scale, more comprehensive studies for the development of instabilities which may induce compression myelopathy and their predictive factors. Therefore, in the current study, we examined the >5-year incidence of myelopathy-inducing cervical spine subluxations in 228 known RA patients. Various predictors were analyzed using multivariable logistic regression. The objective of this study was to elucidate the prognosis of the cervical spine in patients with RA.

## Materials And Methods

### Ethics Statement

This prospective cohort study was conducted at 21 investigation sites (Himeji St. Mary's Hospital, Himeji; Hyogo Prefectural Awaji Hospital, Awaji; Hyogo Prefectural Kakogawa Hospital, Kakogawa; Hyogo Prefectural Nishinomiya Hospital, Nishinomiya; Hyogo Rehabilitation Center, Kobe; Kakogawa City Hospital, Kakogawa; Kanzaki General Hospital, Kanzaki; Kasai City Hospital, Kasai; Kobe Century Memorial Hospital, Kobe; Kobe Medical Center, Kobe; Kobe Rosai Hospital, Kobe; Kobe University Hospital, Kobe; Kohnan Hospital, Kobe; Kohnan Kakogawa Hospital, Kakogawa; Matsubara Mayflower Hospital, Kato; Miki City Hospital, Miki; Miki Sanyo Hospital, Miki; Rokko Island Hospital, Kobe; Saiseikai Hyogoken Hospital, Kobe; Sanda City Hospital, Sanda; and Takasago City Hospital, Takasago, all located in Japan). The study protocol was approved by the institutional review board at each facility. Written informed consent was obtained from each patient. The study was conducted in concordance with the principles of the Declaration of Helsinki and with the laws and regulations of Japan.

### Patients

**Figure 1:**
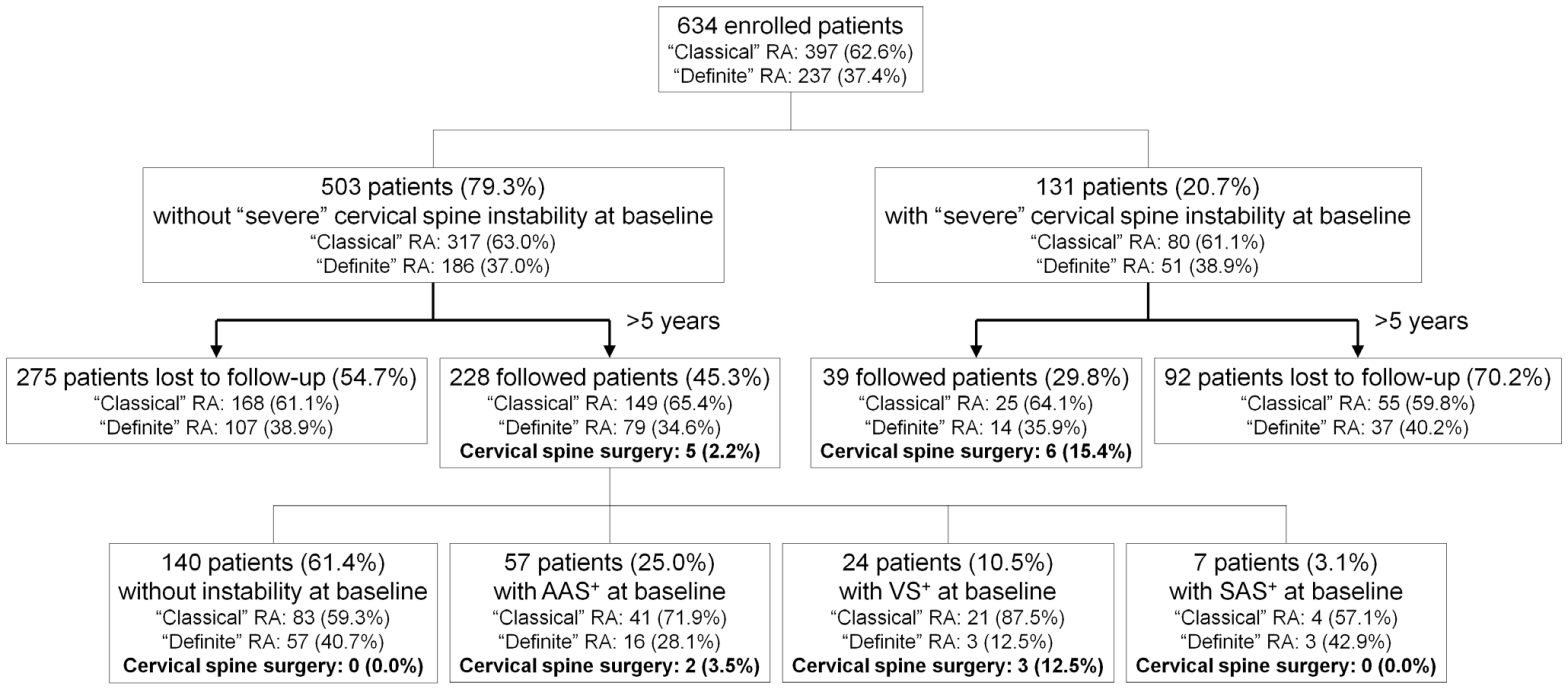
Numbers of patients enrolled, followed, and lost to follow-up with the baseline proportion of “classical” and “definite” rheumatoid arthritis (RA) in the American Rheumatism Association 1958 criteria and the >5-year incidence of cervical spine surgery for myelopathy. Patients were grouped by the type of pre-existing cervical spine involvement: no instability, atlantoaxial subluxation (AAS) alone (shown as AAS^+^), vertical subluxation (VS) without subaxial subluxation (SAS) but with or without AAS (shown as VS^+^), and SAS with and/or without either AAS and/or VS (shown as SAS^+^) and by the level of severity—“moderate” and “severe”.

Between 2001 and 2002, in 21 facilities, 634 outpatients who fulfilled the American Rheumatism Association 1958 criteria for “definite” or “classical” RA [25] and the American College of Rheumatology 1987 revised criteria for RA [26] were enrolled in our study [23], [24]. Cervical radiographs were taken at baseline, and three types of cervical spine instability were categorized into two levels of severity; “moderate” and “severe” criteria were applied as described below. Five hundred and three of 634 cases were identified as patients initially without instability or with “moderate” instability. Between 2006 and 2008, 223 of 503 cases were prospectively followed as outpatients every three months and radiographically reassessed at >5-year follow-up. Additionally, five patients underwent cervical spine surgery for myelopathy during the follow-up period; the last pre-operative radiographs and clinical data were used for evaluation. Therefore, the current study population consisted of the total 228 of 503 patients without any “severe” category of pre-existing cervical spine instability (45.3%) with a mean follow-up period of 6.0±0.8 years. The profile of the cohort and its surgical incidence is shown in **Figure 1**. All data from this study is included in the article.

### Radiographic evaluation

Lateral cervical radiographs, obtained in full-flexion, neutral, and full-extension positions with the standardized protocol (exposure time 80 msec; distance 150 cm; current 250 mA; voltage 72 kV), were used for evaluating cervical spine instability. Cervical radiographs were measured twice at one-week intervals by each of four rheumatologists who were not associated with clinical follow-up of the patients, and the presence of instabilities was determined using the average length of measured values.

Instability was defined as AAS in patients with the anterior atlantodental interval (ADI) >3 mm [3], [4], VS in patients with the Ranawat value <13 mm [12], and SAS in patients with irreducible vertebral translation ≥2 mm without osteophyte formation [15].

“Severe” instability, which indicates impending neurological deficit, was defined as AAS in patients with ADI ≥10 mm [10], VS in patients with Ranawat value ≤10 mm [16], [27], [28], and SAS in patients with irreducible translation ≥4 mm or ≥2 mm at multiple levels [15], [29].

“Moderate” instability was defined in patients with instability who did not meet the “severe” criteria.

Cervical canal stenosis, which is severe enough to induce compression myelopathy, was identified in patients with space available for the spinal cord (SAC) (also called the posterior ADI) ≤13 mm due to “severe” AAS or “severe” VS at C1–C2 level [30] or SAC ≤12 mm due to “severe” SAS at C2–C7 levels [31].

Basilar invagination, which may compress the brainstem and result in life-threatening symptoms, was recognized in patients with the tip of the odontoid process above the McRae line due to “severe” VS [32].

Bilateral hand radiographs were used for classifying the severity of peripheral joint destruction into five categories: Steinbrocker classification stages I–IV [33] and mutilating changes [34], [35]. Mutilating changes were defined in patients with three or more “mutilans fingers [34]” as previously established [35]. Patients with the development of stages I–IV into mutilating changes during the follow-up period were separately categorized. Steinbrocker stages and mutilating changes were decided by accepting the majority decision of three rheumatologists blinded to the research purpose.

### Clinical evaluation

Patient age (<55 [11], [15] and ≥65 [36] years), sex (male [6], [9]), the duration of RA (≥15 years [11]), and previous joint surgery for RA [14], [22], [37], [38] were recorded at baseline. In addition, each patient's follow-up period was compared with the mean duration of 6.0 years.

C-reactive protein (CRP) level was quantified at baseline and assessed whether ≥3.8 mg/dl, a reported average value in RA patients with myelopathic deterioration [39].

Rheumatoid factor (RF) was measured at baseline and positivity was assessed [6], [8], [11], [40].

At baseline, patients received intensive use of disease modifying anti-rheumatic drugs (DMARDs): methotrexate (MTX) (≤8 mg/week), salazosulfapyridine (≤1,000 mg/day), D-penicillamine (≤100 mg/day), or intramuscular gold (≤25 mg/2 weeks). Afterward, MTX, approved for RA since 1999 in Japan, rapidly replaced other DMARDs. Oral corticosteroids were allowed to relieve RA symptoms (≤10 mg/day prednisolone). In more aggressive cases, biologic therapies—infliximab (3 mg/kg/8 weeks), etanercept (10–25 mg/twice a week), adalimumab (40 mg/2 weeks), or tocillizumab (8 mg/kg/4 weeks)—were applied. Patients were categorized based on drug administration at baseline and through more than half of the follow-up period. Since no biologic agent was approved for RA until 2003, patients who had biologic administration for more than half of the follow-up period were similarly identified.

### Statistical analysis

Patients were divided into four groups based on pre-existing cervical spine involvement: no instability, “moderate” AAS alone (shown as AAS^+^), “moderate” VS without SAS but with or without AAS (shown as VS^+^), and “moderate” SAS with and/or without either AAS and/or VS (shown as SAS^+^). To elucidate the influence of baseline instability on the development of cervical spine involvement in RA, the >5-year incidence of the progression of prior instabilities and/or the development of additional instabilities including “severe” instabilities, canal stenosis, and basilar invagination for each group was compared. Distributions of parameters for cervical spine involvement and of RA stages and mutilating changes were compared between baseline and >5-year follow-up. The χ^2^ test, Fisher exact test, paired t-test, or Wilcoxon signed-rank test was used. In addition, intra-class correlation coefficient or κ coefficient was calculated to determine intra- and inter-observer reliabilities for the measurement of radiographic parameters.

Multivariable logistic regression analysis was performed to identify independent predictive risk factors for “severe” instability. Baseline and >5-year differences associated with an increased risk of cervical spine involvement in RA were compared between patients who developed “severe” instabilities versus those who did not; the χ^2^ test, Fisher exact test, Student t-test, or Welch t-test was used. Variables eligible for inclusion in the multivariable models had p values of <0.20 in the univariable analyses and were clinically and/or biologically plausible as described previously [41]. In the multivariable models, the goodness of fit was assessed by the Hosmer-Lemeshow test and the discriminatory ability was assessed by the c-statistic [42]. A random classifier has the c-statistic of 0.5. The c-statistic for a perfect classifier is equal to 1, representing 100% sensitivity (0% false negative rate) and 100% specificity (0% false positive rate). In the present analysis, the false positive rate indicates the percentage of patients with identified predictive factors but without the development of “severe” instabilities. The false negative rate indicates the percentage of patients without the predictors but with “severe” instabilities at >5-year follow-up.

Data analyses were performed with PASW Statistics 18 (SPSS, Chicago, IL). All statistical tests were two-sided. Statistical significance was assessed with p<0.05 and p<0.01. When the incidence of cervical spine lesions was compared between patients with AAS^+^, VS^+^, or SAS^+^ and those without instability at baseline, the Bonferroni adjustment to the threshold for significance was performed; therefore, statistical significance was evaluated with p<0.05/3 = 0.017 and p<0.01/3 = 0.003.

## Results

Baseline radiographic analysis of the cervical spine identified that 228 patients consisted of 140 without any instability, 57 with AAS^+^, 24 with VS^+^, and 7 with SAS^+^. Baseline demographics and disease characteristics of the patients are shown in **Table S1**. There were no significant differences in these variables between 21 investigation sites.

In radiographic measurements, intra-observer reliabilities for parametric ADI, Ranawat value, SAC, and subaxial translation were 0.90–0.94, 0.80–0.85, 0.89–0.95, and 0.81–0.84 by intra-class correlation coefficient. Inter-observer reliabilities were 0.95, 0.90, 0.94, and 0.91. Intra-observer reliabilities for non-parametric basilar invagination and RA stages with mutilating changes were 0.79–0.83 and 0.74–0.79 by κ coefficient. Inter-observer reliabilities were 0.87 and 0.86. All the values indicated an acceptable reproducibility.

### Incidence of cervical spine instabilities and “severe” cervical spine instabilities

To understand the total incidence of cervical spine subluxations, we investigated the incidence of instabilities which consisted of “moderate” and “severe” categories. Then, to clarify the incidence of compression myelopathy-inducing subluxations, we examined the incidence of “severe” instabilities. Forty-three point six percent of 140 patients without cervical spine instability at baseline developed instabilities at >5-year follow-up: AAS in 32.1%, VS in 11.4%, and SAS in 16.4% with some combinations. Furthermore, 12.9% of patients initially without instability presented with “severe” instabilities—“severe” AAS in 3.6%, “severe” VS in 6.4%, and “severe” SAS in 5.0% (**Table 1**).

**Table 1:** Incidence of cervical spine instabilities and “severe” cervical spine instabilities at >5-year follow-up in 228 patients without “severe” cervical spine instability at baseline.

	Cervical spine involvement at baseline				
	No instability (n = 140)	AAS^+^ (n = 57)	VS^+^ (n = 24)	SAS^+^ (n = 7)	Total (n = 228)
Cervical spine involvement at >5-year follow-up					
**Total instability, no. (%)** *	61 (43.6)	57 (100.0)^††^	24 (100.0)^††^	7 (100.0)†	149 (65.4)
“Moderate” or “severe” AAS, no. (%)	45 (32.1)	56 (98.2)^††^	18 (75.0)^††^	4 (57.1)	123 (53.9)
“Moderate” or “severe” VS, no. (%)	16 (11.4)	13 (22.8)^p = 0.041^	24 (100.0)^††^	2 (28.6)	55 (24.1)
“Moderate” or “severe” SAS, no. (%)	23 (16.4)	12 (21.1)	10 (41.7)†	7 (100.0)^††^	52 (22.8)
**“Severe” instability, no. (%)** *	18 (12.9)	19 (33.3)^††^	18 (75.0)^††^	3 (42.9)	58 (25.4)
“Severe” AAS, no. (%)	5 (3.6)	11 (19.3)^††^	3 (12.5)	0 (0.0)	19 (8.3)
“Severe” VS, no. (%)	9 (6.4)	4 (7.0)	17 (70.8)^††^	2 (28.6)	32 (14.0)
“Severe” SAS, no. (%)	7 (5.0)	6 (10.5)	5 (20.8)^p = 0.017^	2 (28.6)	20 (8.8)

Patients were grouped by pre-existing cervical spine involvement: no instability, “moderate” atlantoaxial subluxation (AAS) alone (shown as AAS^+^), “moderate” vertical subluxation (VS) without subaxial subluxation (SAS) but with or without AAS (shown as VS^+^), and “moderate” SAS with and/or without either AAS and/or VS (shown as SAS^+^).

*Including patients fulfilling multiple criteria.

† p<0.05/3 = 0.017, ^††^p<0.01/3 = 0.003 when compared to the incidence in patients initially without instability by the χ^2^ test or Fisher exact test with the Bonferroni adjustment to the threshold for significance.

Fifty-seven patients with AAS^+^ at baseline developed VS in 22.8% at >5-year follow-up, which was higher than those without instability at baseline (11.4%) with a trend toward significance (p = 0.041). Moreover, 33.3% of patients initially with AAS^+^ exhibited “severe” instabilities at a significantly higher rate than that of patients initially without instability (12.9%; p<0.003)—especially “severe” AAS in 19.3% (versus 3.6%; p<0.003) (**Table 1**)

In 24 patients with VS^+^ at baseline, the >5-year incidence of SAS was 41.7%, which was significantly higher than in those without instability at baseline (16.4%; p = 0.010). “Severe” instabilities were developed in 75.0% of patients initially with VS^+^, also more frequently than those initially without instability (12.9%; p<0.003)—notably “severe” VS in 70.8% (versus 6.4%; p<0.003). In addition, the incidence of “severe” SAS in 20.8% of patients with baseline VS^+^ was higher than in those without baseline instability (5.0%), and trended toward significance (p = 0.017) (**Table 1**).

Of seven patients with SAS^+^ at baseline, 42.9% developed “severe” instabilities at >5-year follow-up, which was higher than that of patients without instability at baseline (12.9%) although the comparison did not reach significance (p = 0.061). “Severe” VS occurred in 28.6% of patients initially with SAS^+^ (versus 6.4% of those initially without instability; p = 0.087). “Severe” SAS appeared in 28.6% of patients with baseline SAS^+^ (versus 5.0% of those without baseline instability; p = 0.060) (**Table 1**).

**Figure 2:**
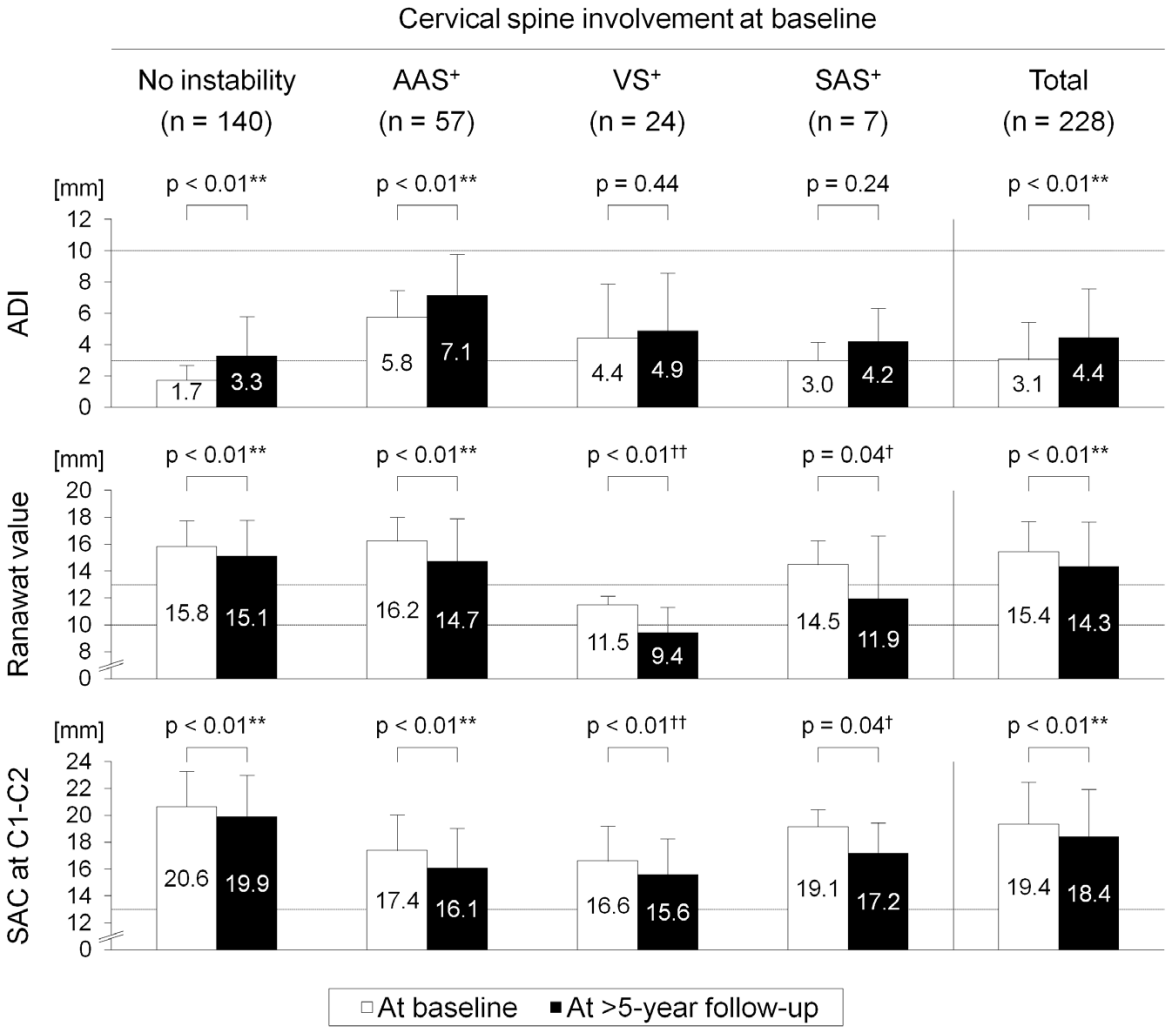
Baseline and >5-year distributions of radiographic parameters for upper cervical spine involvement, the atlantodental interval (ADI), Ranawat value, and space available for the spinal cord (SAC) at C1–C2, in 228 patients without “severe” cervical spine instability at baseline. Patients were grouped by pre-existing cervical spine involvement: no instability, “moderate” atlantoaxial subluxation (AAS) alone (shown as AAS^+^), “moderate” vertical subluxation (VS) without subaxial subluxation (SAS) but with or without AAS (shown as VS^+^), and “moderate” SAS with and/or without either AAS and/or VS (shown as SAS^+^). Data are expressed as mean ± standard deviation. **p<0.01 by the paired t-test. ^†^p<0.05, ^††^p<0.01 by the Wilcoxon signed-rank test because of the small number of cases.

In distributions of radiographic parameters, the ADI significantly increased during >5 years only in patients initially without instability and those with AAS^+^ (p<0.01 for both groups). The Ranawat value decreased in all patient groups (p<0.01 for patients without instability, with AAS^+^, and with VS^+^ and p = 0.04 for those with SAS^+^) (**Figure 2**).

### Incidence of cervical canal stenosis and basilar invagination

While 4.3% of patients without baseline instability developed cervical canal stenosis with decreased SAC (SAC ≤13 mm at C1–C2 in 2.9% and SAC ≤12 mm at C2–C7 in 2.1%), 15.8% of those with baseline AAS^+^ exhibited stenosis (p = 0.014)—particularly SAC ≤13 mm at C1–C2 in 10.5% (p = 0.036). Patients with baseline VS^+^ presented with cervical stenosis in 16.7% (p = 0.041) who all showed SAC ≤13 mm at C1–C2 (p = 0.017). Patients with baseline SAS^+^ developed stenosis in 14.3% (p = 0.295) who demonstrated SAC ≤12 mm at C2–C7 (p = 0.179) (**Table 2**).

**Table 2:** Incidence of cervical canal stenosis and basilar invagination at >5-year follow-up in 228 patients without “severe” cervical spine instability at baseline.

	Cervical spine involvement at baseline				
	No instability (n = 140)	AAS^+^ (n = 57)	VS^+^ (n = 24)	SAS^+^ (n = 7)	Total (n = 228)
Cervical spine involvement at >5-year follow-up					
**Cervical canal stenosis, no. (%)** *	6 (4.3)	9 (15.8)†	4 (16.7)^p = 0.041^	1 (14.3)	20 (8.8)
SAC ≤13 mm at C1–C2, no. (%)	4 (2.9)	6 (10.5)^p = 0.036^	4 (16.7)^p = 0.017^	0 (0.0)	14 (6.1)
SAC ≤12 mm at C2–C7, no. (%)	3 (2.1)	4 (7.0)	2 (8.3)	1 (14.3)	10 (4.4)
**Basilar invagination, no. (%)**	4 (2.9)	3 (5.3)	8 (33.3)^††^	0 (0.0)	26 (11.4)
**Cervical canal stenosis and/or basilar invagination, no. (%)** *	10 (7.1)	10 (17.5)^p = 0.028^	9 (37.5)^††^	1 (14.3)	30 (13.2)

Patients were grouped by pre-existing cervical spine involvement: no instability, “moderate” atlantoaxial subluxation (AAS) alone (shown as AAS^+^), “moderate” vertical subluxation (VS) without subaxial subluxation (SAS) but with or without AAS (shown as VS^+^), and “moderate” SAS with and/or without either AAS and/or VS (shown as SAS^+^).

*Including patients fulfilling multiple criteria.

† p<0.05/3 = 0.017, ^††^p<0.01/3 = 0.003 when compared to the incidence in patients initially without instability by the χ^2^ test or Fisher exact test with the Bonferroni adjustment to the threshold for significance.

SAC, space available for the spinal cord.

Basilar invagination with cranial migration of the odontoid tip was detected in 2.9% of patients initially without instability whereas it was found in 33.3% of those initially with VS^+^ (p<0.003) and 5.3% of those with AAS^+^ (p = 0.415) (**Table 2**).

Therefore, the >5-year incidence of cervical canal stenosis and/or basilar invagination, which may fully progress to myelopathy and/or brainstem symptoms, was higher in patients with baseline instability (17.5% in those with AAS^+^, 37.5% in those with VS^+^, and 14.3% in those with SAS^+^) than in patients without instability (7.1%; p = 0.028, p<0.003, and p = 0.427, respectively) (**Table 2**).

In distributions of radiographic parameters, the SAC at C1–C2 significantly decreased during >5 years in every patient group (p<0.01 for patients without instability, with AAS^+^, and with VS^+^ and p = 0.04 for those with SAS^+^) (**Figure 2**).

### Predictive risk factors for the development of “severe” cervical spine instabilities

Radiographic cervical spine analysis at baseline and at >5-year follow-up identified the development of “severe” instabilities in 58 of 228 patients (25.4%), as summarized in **Table 1**. Radiographic peripheral assessment disclosed a significant shift in the distribution of RA stages and mutilating changes (p<0.01). Patients with stage I or II decreased from 49 (21.5%) to 18 (7.9%; p<0.01) while those with stage III or IV increased from 162 (71.1%) to 182 (79.8%; p = 0.03) and those with mutilating changes increased from 17 (7.5%) to 28 (12.3%; p = 0.08). Eleven patients (4.8%) initially with stages I–IV developed mutilating changes during the follow-up period. Baseline and >5-year demographics and disease characteristics in patients who developed “severe” instabilities and those who did not develop are shown in **Table 3**.

**Table 3:** Baseline and >5-year demographics and disease characteristics in 228 patients with and without “severe” cervical spine instability at >5-year follow-up.

	Patients with “severe” cervical spine instability (n = 58)	Patients without “severe” cervical spine instability (n = 170)	p Value*
**At baseline**			
**Demographics and clinical characteristics**			
Age, mean ± SD years	61.4±9.3	60.6±10.8	0.62
<55, no. (%)	17 (29.3)	50 (29.4)	0.80
55–64, no. (%)	15 (25.9.)	51 (30.0)	
≥65, no. (%)	26 (44.8)	69 (40.6)	
Male sex, no. (%)	11 (19.0)	34 (20.0)	0.86
RA duration, mean ± SD years	14.0±9.5	12.9±11.0	0.52
≥15, no. (%)	21 (36.2)	47 (27.6)	0.22
**Previous joint surgery, no. (%)**	36 (62.1)	60 (35.3)	<0.01^††^
CRP, mean ± SD mg/dl	2.5±1.9	1.5±1.8	<0.01^††^
≥3.8, no. (%)	14 (24.1)	20 (11.8)	0.02†
RF positive, no. (%)	50 (86.2)	131 (77.1)	0.14
**Medications**			
Corticosteroids, no. (%)	49 (84.5)	89 (52.4)	<0.01^††^
MTX, no. (%)	30 (51.7)	78 (45.9)	0.44
Other DMARDs, no. (%)	28 (48.3)	90 (52.9)	0.54
**RA stages and mutilating changes**			
Stage I or II, no. (%)	4 (6.9)	45 (26.5)	<0.01^††^
Stage III or IV, no. (%)	43 (74.1)	119 (70.0)	
Mutilating changes, no. (%)	11 (19.0)	6 (3.5)	
**At >5-year follow-up**			
**Demographics and clinical characteristics**			
Follow-up period, mean ± SD years	5.9±1.2	6.1±0.6	0.371
≥6.0, no. (%)	30 (51.7%)	96 (56.5%)	0.53
**Medications**			
Biologic agents, no. (%)	4 (6.9)	6 (3.5)	0.28
**RA stages and mutilating changes**			
Development of stages I–IV into mutilating changes, no. (%)	7 (12.1)	4 (2.4)	<0.01^††^

*Tested by the χ^2^ test, Fisher exact test, Student t-test, or Welch t-test.

† p<0.05. ^††^p<0.01.

CRP, C-reactive protein; DMARD, disease modifying anti-rheumatic drug; MTX, methotrexate; RA, rheumatoid arthritis; RF, rheumatoid factor; SD, standard deviation.

In the univariable analysis, previous joint surgery, CRP ≥3.8 mg/dl, corticosteroid administration, Steinbrocker stage III or IV at baseline (without the development of mutilating changes during the follow-up period), mutilating changes at baseline, and the development of stages I–IV into mutilating changes during the follow-up period were statistically significant for the development of “severe” cervical spine instabilities at >5-year follow-up (p<0.05) (**Table 4**). Rheumatoid factor positive was not significant for “severe” instability with p of <0.20. Methotrexate, other DMARDs, and biologic agent administration was not statistically significant with p of ≥0.20 but was thought to be clinically and biologically relevant in the progression of rheumatoid involvement.

**Table 4:** Odds ratios (ORs), 95% confidence intervals (95% CIs), and p values for “severe” cervical spine instability by univariable and multivariable logistic regression analyses.

	Univariable analysis		Multivariable analysis*		Stepwise multivariable analysis**	
	OR (95% CI)	p Value	Adjusted OR (95% CI)	p Value	Adjusted OR (95% CI)	p Value
**Demographics and clinical characteristics**						
<55 years old	1.16 (0.52–2.56)	0.72	Not included		Not included	
≥65 years old	1.28 (0.62–2.66)	0.51	Not included		Not included	
Male sex	0.94 (0.44–1.99)	0.86	Not included		Not included	
RA duration ≥15 years	1.49 (0.79–2.80)	0.22	Not included		Not included	
Previous joint surgery	3.00 (1.62–5.56)	<0.01^††^	1.93 (0.95–3.90)	0.07	1.89 (0.96–3.75)	0.07
CRP ≥3.8 mg/dl	2.39 (1.11–5.11)	0.03†	1.37 (0.57–3.27)	0.48	Not included	
RF positive	1.86 (0.81–4.26)	0.14	1.26 (0.50–3.14)	0.62	Not included	
Follow-up period ≥6.0 years	0.83 (0.45–1.50)	0.53	Not included		Not included	
**Medications**						
Corticosteroids	4.96 (2.29–10.72)	<0.01^††^	4.65 (1.98–10.91)	<0.01^††^	4.57 (1.98–10.53)	<0.01^††^
MTX	1.26 (0.70–2.30)	0.44	0.70 (0.30–1.63)	0.41	Not included	
Other DMARDs	0.83 (0.46–1.51)	0.54	0.81 (0.36–1.82)	0.62	Not included	
Biologic agents	2.02 (0.55–7.44)	0.29	1.04 (0.23–4.60)	0.96	Not included	
**RA stages and mutilating changes**						
Stage III or IV at baseline (without the development of mutilating changes)	4.83 (1.42–16.45)	0.012†	3.65 (1.02–13.13)	0.047†	3.92 (1.11–13.81)	0.03†
Mutilating changes at baseline	27.50 (5.93–127.60)	<0.01^††^	21.23 (3.97–113.57)	<0.01^††^	23.29 (4.51–120.34)	<0.01^††^
Development of stages I–IV into mutilating changes	26.25 (4.82–143.06)	<0.01^††^	13.44 (2.24–80.55)	<0.01^††^	14.65 (2.56–83.88)	<0.01^††^

*The Hosmer-Lemeshow goodness-of-fit χ^2^ p = 0.85 (8 degrees of freedom) and the c-statistic for the model  = 0.80.

**The Hosmer-Lemeshow goodness-of-fit χ^2^ p = 0.93 (7 degrees of freedom) and the c-statistic for the model  = 0.79.

† p<0.05. ^††^p<0.01.

CRP, C-reactive protein; DMARD, disease modifying anti-rheumatic drug; MTX, methotrexate; RA, rheumatoid arthritis; RF, rheumatoid factor.

In the multivariable analysis, we established a multivariable logistic regression model including the variables described above (**Table 4**). This multivariable model identified four variables as significant predictive risk factors for the progression to “severe” instability: corticosteroid administration (odds ratio [OR] 4.65, 95% confidence interval [95% CI] 1.98–10.91, p<0.01), Steinbrocker stage III or IV at baseline (OR 3.65, 95% CI 1.02–13.13, p = 0.047), mutilating changes at baseline (OR 21.23, 95% CI 3.97–113.57, p<0.01), and the development of stages I–IV into mutilating changes during the follow-up period (OR 13.44, 95% CI 2.24–80.55, p<0.01). The high ORs of pre-existing mutilating changes, new development of mutilating changes, and then established Steinbrocker stage III or IV suggest a direct association of the severity of peripheral erosions with the development of cervical spine instabilities in RA patients. Accordingly, the high OR of the administration of corticosteroids could be explained by concomitant severe disease activity. Previous joint surgery was not significant but showed a weak correlation (OR 1.93, 95% CI 0.95–3.90, p = 0.07). As the ORs of the presence of surgically treated joints, high CRP, and positive RF were modest, these factors would be supportive, but not strong enough to estimate RA severity in the cervical spine. The administration of MTX, other DMARDs, and biologic agents did not show a strong association with the development of “severe” instabilities in this study. This model had a good predictive ability, with the c-statistic of 0.80.

To reduce the risk for overfitting variables [43], we designed an additional multivariable model based on backward stepwise variable selection (**Table 4**). This multivariable model also detected the same four significant variables: corticosteroid administration (OR 4.57, 95% CI 1.98–10.53, p<0.01), stage III or IV at baseline (OR 3.92, 95% CI 1.11–13.81, p = 0.03), mutilating changes at baseline (OR 23.29, 95% CI 4.51–120.34, p<0.01), and the development of stages I–IV into mutilating changes during the follow-up period (OR 14.65, 95% CI 2.56–83.88, p<0.01). Previous joint surgery was marginally significant (OR 1.89, 95% CI 0.96–3.75, p = 0.07). This model also had an acceptable predictive ability, with the c-statistic of 0.79.

## Discussion

In the current study, we assessed and compared the >5-year incidence of cervical spine instabilities with impending neurological deficit in 228 established RA patients with and without baseline instability, which demonstrated that the development of these serious lesions was accelerated in response to the type of baseline cervical spine instability—especially VS. Multivariable logistic regression analysis identified advanced peripheral erosiveness, concomitant corticosteroid treatment, and marginally previous joint surgery as independent risk factors for the development of severe cervical spine instabilities in RA. This study clarifies these clinically important findings with statistically robust, more conclusive evidence, which provides a comprehensive understanding of the progression of cervical spine involvement in patients with RA.

The incidence of cervical spine instabilities depends on the diagnostic criteria for subluxation and the severity of RA. In this study, we used previously validated criteria [24] and enrolled patients with “definite” or “classical” RA only. During 6.0±0.8 years, 43.6% of patients without pre-existing cervical spine instability developed instabilities. Patients initially without instability further developed “severe” instabilities in 12.9% and canal stenosis and/or basilar invagination in 7.1%. The detailed occurrence rate and pattern of each lesion were described in our previous report [24]. The observed percentage of patients who developed instabilities at endpoint is comparable to the previously reported incidence of instabilities in patients with long-standing RA (46.7%) in a mean 6.1-year follow-up study [17]. This supports that our diagnostic criteria and radiographic measurement are acceptable for the assessment of patients with known RA. In the current study, in order to elucidate the progression of cervical spine involvement, we designed a comparative cohort study for the incidence of instabilities in established RA patients with and without pre-existing instability.

“Severe” cervical spine instabilities occurred in 33.3–75.0% of patients with baseline “moderate” instability, while these lesions only appeared in 12.9% of patients initially without instability. In patients initially with AAS^+^, 19.3% progressed to “severe” AAS and 22.8% developed VS. Notably, in patients initially with VS^+^, 70.8% progressed to “severe” VS, 41.7% newly developed SAS, and 20.8% further developed “severe” SAS. Patients initially with SAS^+^ also frequently had the progression of instabilities, although no statistical significance was obtained due to the small number of cases. Additionally, distributions of radiographic parameters support this higher progression tendency of instabilities in patients with initial instability. These results indicate accelerated development of cervical spine involvement in RA patients with pre-existing instability—especially VS.

Cervical canal stenosis and/or basilar invagination occurred in 14.3–37.5% of patients with baseline “moderate” instability, while these complications only appeared in 7.1% of patients initially without instability. The highest incidence of both these lesions was detected in patients initially with VS^+^. In our previous study, we found that patients with pre-existing VS and/or SAS had substantially worse cervical spine instabilities [23]. The current analysis further demonstrates that patients with pre-existing VS are highly likely to develop myelopathy and/or brainstem symptoms, which is similar to other studies [8]–[10], [17], [22], [27]–[30]. This study lacks data relating RA and neurological symptoms due to the difficulties in standardizing assessment between multiple centers. However, the observed incidence of cervical spine surgery may suggest neurological deterioration. Surgery was most frequently seen in patients initially with VS^+^. The development of cervical canal stenosis and/or basilar invagination, severe enough to induce spinal cord and/or brainstem compression, are accelerated in patients with pre-existing instability—especially VS.

Recently, advances in RA therapies have drastically changed the clinical course even in patients with active disease [44], and may require careful interpretation of our results in treated populations for its effect on incidence. The impact of biologic therapies on cervical spine instability in RA is still poorly understood. A mean 4.4-year follow-up study of patients with the compliant >2-year administration of MTX and biologic agents demonstrated that only 8.3% without baseline instability developed instabilities while 80.8% with baseline instability had the progression of prior instabilities and/or development of additional instabilities [45]. A similar finding was observed in hip, knee, and ankle joints [46]; under biologic therapies, the development of radiographic damage was suppressed in joints with baseline Larsen grades 0 to II [47] but damaged joints with baseline Larsen grades III and IV showed further progression. These lines of evidence suggest potential effectiveness of intensive biologic therapies on preventing the development of cervical spine involvement in the early course of RA rather than on reducing the progression of pre-existing instabilities with the structural damage of the joints, bones, and ligaments. Also, this supports the early initiation of biologic therapies for the management of RA, corresponding with the European League Against Rheumatism recommendations [48]. Further studies need to be conducted.

Reported evidence indicates that patients with “severe” cervical spine instabilities, narrowed spinal canals, and/or cranial migration of the odontoid process are at risk for irreversible neurological damage [9], [10], [15], [17], [27]–[31]. The soft-tissue pannus as well as bone can induce symptomatic compression of the spinal cord in patients with RA [30]. In the present study, intra-observer reliabilities for the Ranawat value and subaxial translation were relatively low. Variations in radiographic technique and evaluation can easily affect the accuracy and precision of these radiographic measurements, which are more challenging to measure in RA patients because of generalized osteopenia, multiple subluxations, and erosions [12], [14], [17]. Therefore, RA patients with these advanced cervical spine lesions should receive not only radiographic but also magnetic resonance imaging examinations in the cervical spine.

Our multivariable analysis identified independent predictive risk factors for the development of “severe” instabilities: corticosteroid administration, Steinbrocker stage III or IV at baseline, mutilating changes at baseline, and the development of stages I–IV into mutilating changes during the follow-up period. These four variables, with significance in all the univariable, multivariable, and stepwise multivariable analyses, are relatively robust predictors for the progression of cervical spine involvement in RA. In addition, the consistent results from these analyses support further validation of the clinically-led multivariable model before applying stepwise approach.

Prolonged and high dose administration of corticosteroids is shown to aggravate cervical subluxations in RA patients [5], [6], [8], [15], [49], [50]. Increased incidence of SAS, directly related to the duration of corticosteroid therapy, is reported even in non-RA patients [51]. Our results also indicate a negative effect of corticosteroids on the cervical spine in RA; however, we cannot conclude whether it is explained by concomitant severe disease activity or by corticosteroid-mediated structural damage. Biologically, corticosteroids induce the loss of bone mineral density [52] and articular chondrocyte death [53]. Further investigations for possible pathomechanisms are needed.

This study, which began in 2001, detected no obvious therapeutic effect of MTX and biologic therapies. However, their ineffectiveness cannot be concluded because their approval occurred in 1999 and 2003, respectively, and our patients had long RA histories predating these treatments. In addition, the dosages of medications used were lower than those used in Western countries. Reduced effectiveness of RA therapies indicates our results should be carefully interpreted.

Mutilating changes are reported to highly correlate with aggressive cervical instabilities in RA [22], [35], [39], [40], [50]. A prior multivariable analysis further demonstrated that patients with ≥10% peripheral joint damage at 5 years were 15.9 times more likely to develop AAS at 8–13 years than those with <10% [36]. In the context of this evidence, our multivariable models indicate that, in addition to patients with established mutilans, those without mutilans have the potential to develop severe cervical spine involvement with simultaneous development of their peripheral erosiveness into mutilans.

Steinbrocker stage III or IV was also statistically significant for the progression to “severe” instability. Previous joint surgery was a marginally significant predictor; a high prevalence of cervical spine instabilities in RA patients undergoing joint surgery is often reported [14], [22], [37], [38]. Based on the ORs and 95% CIs of these variables, we propose that RA patients not only with mutilating changes but also with stage III or IV and severe disease activity—long-term corticosteroid administration and/or previous joint surgery—should receive radiological follow-up in the cervical spine.

This study is limited in that the duration of RA ranged widely. The currently used classification system for the severity of RA might be a somewhat obsolete and insensitive method of assessment. The biggest limitation is a low follow-up rate of 45.3%. As shown in **Table S1**, followed patients were younger than patients lost to follow-up. In addition, patients with baseline “severe” instability, speculated to have worse conditions, showed a lower follow-up rate. This indicates that many older, advanced RA outpatients potentially drop out due to polyarthralgia, worsening general condition, and/or cervical problems. Furthermore, patients who developed “severe” instabilities during the follow-up period might be lost more frequently, implying that the endpoint incidence of “severe” instabilities is higher than the observed data suggests. Taken together, patients' loss to follow-up is likely not completely random in this study.

Survival analyses such as the Cox proportional hazards analysis should provide more useful information for identifying predictive factors for “severe” instability when cervical radiographs obtained at multiple time points are available. The mixed-effects model may also be more suitable in the case of data missing at random between patients with and without the development of “severe” instabilities. The application of these statistical analysis methods is an issue to be considered in future studies.

## Conclusions

This prospective cohort study demonstrates accelerated development of cervical spine involvement in RA patients with pre-existing instability—especially VS. Established mutilating changes and progressive development of non-mutilating into mutilating changes strongly indicate poor prognosis of the cervical spine in patients with RA. Steinbrocker stage III or IV, corticosteroid treatment, and possibly previous joint surgery also correlate with the progression of cervical spine instabilities in RA.

## Supporting Information

Table S1 **Baseline demographics and disease characteristics in 634 enrolled patients.**
(PDF)
