# Supplementary material for: Accelerated Development of Cervical Spine Instabilities in Rheumatoid Arthritis: A Prospective Minimum 5-Year Cohort Study
Source: PLoS One. 2014 Feb 18;9(2):e88970. doi: 10.1371/journal.pone.0088970 (PMC3928338; doi:10.1371/journal.pone.0088970)
Supplement: Table S1 — Baseline demographics and disease characteristics in 634 enrolled patients. (PDF) [file pone.0088970.s001.pdf]

Table S1. Baseline demographics and disease characteristics in 634 enrolled patients.

	Patients without "severe" cervical spine instability at baseline							Patients with "severe" cervical spine instability at baseline			Total
	Followed patients					Patients lost to follow-up	Total	Followed patients	Patients lost to follow-up	Total	
	Cervical spine involvement at baseline										
	No instability	AAS$^+$	VS$^+$	SAS$^+$	Total						
	(n = 140)	(n = 57)	(n = 24)	(n = 7)	(n = 228)	(n = 275)	(n = 503)	(n = 39)	(n = 92)	(n = 131)	(n = 634)
**Demographics and clinical characteristics**											
Age, mean ± SD years	62.3 ± 9.9	56.9 ± 11.1	59.3 ± 9.6	67.7 ± 8.0	60.8 ± 10.4	63.7 ± 12.0	62.4 ± 11.4	61.7 ± 8.4	66.7 ± 10.3	65.2 ± 10.0	63.0 ± 11.2
Sex, no. of male (%)	33 (23.6)	10 (17.5)	1 (4.2)	1 (14.3)	45 (19.7)	52 (18.9)	97 (19.3)	2 (5.1)	21 (22.8)	23 (17.6)	120 (18.9)
RA duration, mean ± SD years	12.6 ± 11.1	13.8 ± 9.0	13.6 ± 9.2	18.3 ± 14.6	13.2 ± 10.5	11.0 ± 9.3	12.0 ± 9.9	18.0 ± 9.0	18.0 ± 10.7	18.0 ± 10.2	13.3 ± 10.3
Previous joint surgery, no. (%)	54 (38.6)	26 (45.6)	14 (58.3)	2 (28.6)	96 (42.1)	114 (41.5)	210 (41.7)	32 (82.1)	61 (66.3)	93 (71.0)	303 (47.8)
CRP, mean ± SD mg/dl	1.5 ± 1.7	2.1 ± 2.2	2.4 ± 1.9	2.0 ± 2.1	1.8 ± 1.9	2.2 ± 2.8	2.0 ± 2.4	1.9 ± 1.9	2.2 ± 2.1	2.1 ± 2.0	2.0 ± 2.3
RF positive, no. (%)	114 (81.4)	44 (77.2)	17 (70.8)	6 (85.7)	181 (79.4)	226 (82.2)	407 (80.9)	31 (79.5)	75 (81.5)	106 (80.9)	513 (80.9)
**Medications**											
Corticosteroids, no. (%)	72 (51.4)	43 (75.4)	18 (75.0)	5 (71.4)	138 (60.5)	160 (58.2)	298 (59.2)	27 (69.2)	71 (77.2)	98 (74.8)	396 (62.5)
MTX, no. (%)	61 (43.6)	32 (56.1)	12 (50.0)	3 (42.9)	108 (47.4)	117 (42.5)	225 (44.7)	20 (51.3)	35 (38.0)	55 (42.0)	280 (44.2)
Other DMARDs, no. (%)	77 (55.0)	24 (42.1)	14 (58.3)	3 (42.9)	118 (51.8)	148 (53.8)	266 (52.9)	16 (41.0)	53 (57.6)	69 (52.7)	335 (52.8)
**RA stages and mutilating changes**											
Stage I or II, no. (%)	40 (28.6)	8 (14.0)	0 (0.0)	1 (14.3)	49 (21.5)	85 (30.9)	134 (26.6)	0 (0.0)	7 (7.6)	7 (5.3)	141 (22.2)
Stage III or IV, no. (%)	96 (68.6)	46 (80.7)	16 (66.7)	4 (57.1)	162 (71.1)	163 (59.3)	325 (64.6)	28 (71.8)	60 (65.2)	88 (67.2)	413 (65.1)
Mutilating changes, no. (%)	4 (2.9)	3 (5.3)	8 (33.3)	2 (28.6)	17 (7.5)	27 (9.8)	44 (8.7)	11 (28.2)	25 (27.2)	36 (27.5)	80 (12.6)

Patients were grouped by the type of pre-existing cervical spine involvement: no instability, atlantoaxial subluxation (AAS) alone (shown as AAS$^+$), vertical subluxation (VS) without subaxial subluxation (SAS) but with or without AAS (shown as VS$^+$), and SAS with and/or without either AAS and/or VS (shown as SAS$^+$) and by the level of severity—"moderate" and "severe".

CRP, C-reactive protein; DMARD, disease modifying anti-rheumatic drug; MTX, methotrexate; RA, rheumatoid arthritis; RF, rheumatoid factor; SD, standard deviation.
